# Science, Dualities and the Phenomenological Map

**DOI:** 10.1007/s10699-022-09850-4

**Published:** 2022-06-07

**Authors:** H. G. Solari, M. A. Natiello

**Affiliations:** 1 Departamento de Física, FCEN-UBA and IFIBA-CONICET, Buenos Aires, Argentina; 2 Centre for Mathematical Sciences, Lund University, Lund, Sweden

**Keywords:** Abduction, Pragmaticism, Bild, Epistemological shift

## Abstract

We present an epistemological schema of natural sciences inspired by Peirce’s pragmaticist view, stressing the role of the *phenomenological map*, that connects reality and our ideas about it. The schema has a recognisable mathematical/logical structure which allows to explore some of its consequences. We show that seemingly independent principles as the requirement of reproducibility of experiments and the Principle of Sufficient Reason are both implied by the schema, as well as Popper’s concept of falsifiability. We show that the schema has some power in demarcating science by first comparing with an alternative schema advanced during the first part of the 20th century which has its roots in Hertz and has been developed by Einstein and Popper. Further, the identified differences allow us to focus in the construction of Special Relativity, showing that it uses an intuited concept of velocity that does not satisfy the requirements of reality in Peirce. While the main mathematical observation connected with this issue has been known for more than a century, it has not been investigated from an epistemological point of view. A probable reason could be that the socially dominating epistemology in physics does not encourage such line of work. We briefly discuss the relation of the abduction process presented in this work with discussions regarding “abduction” in the literature and its relation with “analogy”.

## Introduction

Ever since the Greeks attempted to conceive an understanding of the world, a duality—a relation of two worlds—has been in the centre of the scene. In various Socratic dialogues [e.g., (Plato, 2014)], Plato refers to the world of forms, or ideas, an eternal world of perfection, as well as to the world of imperfect copies, our material world. Plato’s theory of forms is well known and has deserved extensive discussion^1^. We will simply observe that the duality it introduces has been a substantial part of epistemology since then. We shall call the world of forms “*Ideal World*” (IW) and the material world, the world accessible with our senses, “*Sensible World*” (SW). We credit Galileo for being one of the first in advancing that the IW was populated by a mental operation he called *idealisation* that produced perfect^2^ (or at least perfected) models of the observable (Galilei, 1914). Several authors have worked along this conception. One of the most remarkable has been (Husserl, 1983) who used the term *ideation* to indicate the process by which the observable was incorporated in our perception as ideas. Piaget and García (1989) made a clear distinction between the observed, that what reaches our senses, and the *facts*, the ideated, that what is incorporated in our knowledge as perceived. All these authors have in common not only the duality between both worlds, but the existence of correspondences between elements in one and the other world. Plato emphasised the relation $IW{\mathop {\longmapsto }\limits ^{\Gamma }}SW$ while Galileo stressed the inverse relation $SW{\mathop {\longmapsto }\limits ^{\varPi }}IW$. We shall call the pair $\left( \varPi ,\Gamma \right)$ the *phenomenological map*. As far as we know, the properties and the consequences regarding the assumed existence of a phenomenological map have received little attention in the past. The matter was considered in Margenau and Mould (1957) and (Dingle, 1960b), who referred to the phenomenological map as “rules of correspondence” but they did not advance into the implied logical structure. In turn, Feigl^3^ reminds us in his analysis of the “orthodox view of theories”:

In the picturesque but illuminating elucidations used, e.g., by Schlick, Carnap, Hempel, and Margenau, the ”pure calculus”, i.e., the uninterpreted postulate system, ”floats” or ”hovers” freely above the plane of empirical facts. It is only through the ”connecting links,” i.e., the ”coordinative definitions” (Reichenbach’s terms, roughly synonymous with the ”correspondence rules” of Margenau and Carnap, or the ”epistemic correlations” of Northrop, and only related to but not strictly identical with Bridgman’s ”operational definitions” ), that the postulate system acquires empirical meaning.

and proposes a more strict correspondence, that he names “bridge laws”:

Let me emphasize once more that this manner of regarding theories is a matter of highly artificial reconstruction. It does not in the least reflect the way in which theories originate. Correspondence rules thus understood differ from bridge laws in that the latter make empirical assertions.

In the present work we intend to show that the phenomenological map is a key element in a traditional conception of science. Its construction implies foundational reasoning principles such as Leibniz’ Principle of Sufficient Reason [see e.g., (Ballard, 1960)] and the related No Arbitrariness Principle (NAP) (Solari & Natiello, 2018). We will show that other conceptions of science, such as that put forward by Einstein (1936, 1940) and (Popper, 1959) go back to the platonic view, in the sense that only the map $\Gamma$ (the interpretation) is considered, and because of this, they are forced to introduce an independent symmetry principle (somehow borrowed from the traditional conception). Further, we show that within the present framework the map $\varPi$ cannot be completed for Special Relativity (SR), which results then in a theory that depends on previous epistemic decisions. Finally, we contrast abduction and analogical thinking as a basis for constructing theories of Nature.

## A Pragmaticist View of the Traditional^4^ Concept of Science

Charles Peirce found convenient to rename his philosophical standpoint from pragmatism into pragmaticism, his argument being:

So then, the writer, finding his bantling ”pragmatism” so promoted, feels that it is time to kiss his child good-by and relinquish it to its higher destiny; while to serve the precise purpose of expressing the original definition, he begs to announce the birth of the word ”pragmaticism,” which is ugly enough to be safe from kidnappers.(Peirce, 1994, CP 5.414)

We will try to keep our view within Peirce’s original view, knowing that every reader is a potential kidnapper of the term (the present authors included).

Peirce introduced the fundamental concept of **Reality** as follows:

“Such is the method of science. Its fundamental hypothesis, restated in more familiar language, is this: There are Real things, whose characters are entirely independent of our opinions about them; those Reals affect our senses according to regular laws, and, though our sensations are as different as are our relations to the objects, yet, by taking advantage of the laws of perception, we can ascertain by reasoning how things really and truly are; and any man, if he have sufficient experience and he reason enough about it, will be led to the one True conclusion. The new conception here involved is that of Reality.” (Peirce, 1994, CP 5.384)

Since reality is independent of the subject we can say that a fundamental requirement of the real is to be objective or at least intersubjective. While the observations are prone of circumstances such as where and when, as well as the observer, the *facts *have been usually deprived of such elements. The *ideated facts* are the reality in Peirce, and these facts are the point upon which two observers can agree (Peirce, 1955, p. 150). We also learn about hypothesis in Peirce, although we prefer to use the name **conjectures**:

A hypothesis is something which looks as if it might be true and were true, and which is capable of verification or refutation by comparison with facts. The best hypothesis, in the sense of the one most recommending itself to the inquirer, is the one which can be the most readily refuted if it is false. (Peirce, 1994, CP 1.120)

**Fig. 1:**
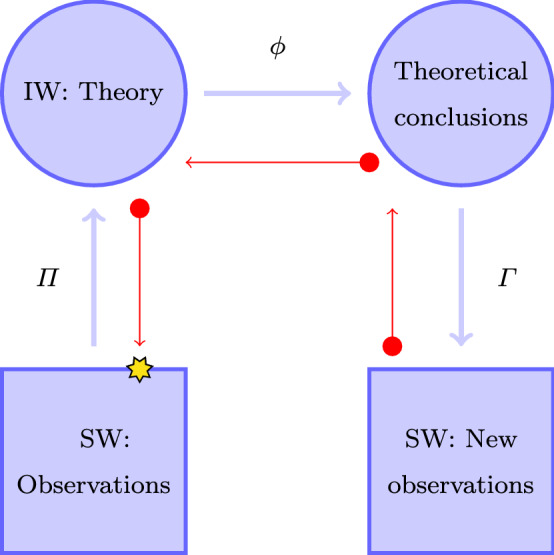
The proposed schema of Science. $\varPi$ is a projection that produces the real out of the observed, $\phi$ stands for a theoretical elaboration (which eventually can be none, in such a case $\phi$ is the identity *Id*) and $\Gamma$ is the interpretation that produces an expected observation

For Peirce, predictions are predictions of facts, since events/observations are haphazard (Peirce, 1955, pp. 153, 214). We will take a compatible but alternative view: we shall call **prediction** an expected observation based upon the known facts, the hypothesis *h* (conjectures), and logical/deductive elaborations. To produce a prediction we have to elaborate our known facts and, before verification, we have to provide the **particularities**^5^(unpredicted elements) that move us back to SW from the IW. In mathematics, the map $\varPi$ would be called a projection, and the predictive mapping, $\Gamma$, is named a “lift”,^6^ while we will use the symbol $\phi$ to denote the rational elaboration of ideas. When dealing with spontaneous observations, the need for $\Gamma$ may appear unjustified, but if we are to conceive experiments to test a theory, $\Gamma$ is a most relevant object that tells us what to expect, and it is this expectation what is really confronted against experimental results.^7^ Figure 1 shows this outlined schema of science.

There are three conditions to be satisfied for the schema $\left\{ \varPi ,\phi ,\Gamma \right\}$ to be consistent. If we ideate a set of particular observations –name the particularism by $\alpha$– to construct a theory, $\varPi (\left\{ Obs\right\} _{\alpha })$, when we interpret the ideas in the theory using the same particularism, $\Gamma _{\alpha }\left( \varPi (\left\{ Obs\right\} _{\alpha }\right)$, we must recover the observed,

1 $$\begin{aligned} \Gamma _{\alpha }\left( \varPi (\left\{ Obs\right\} _{\alpha })\right) =\left\{ Obs\right\} _{\alpha } \end{aligned}$$

Thus, $\Gamma _{\alpha }\circ \varPi$ acts as the identity within particularism $\alpha$. Correspondingly, if we produce the theory out of an interpreted set of concepts and relations, $\varPi \left( \Gamma _{\alpha }(\tau )\right)$, we should get the original set of concepts and relations $\tau$ constituting the theory

2 $$\begin{aligned} \varPi \left( \Gamma _{\alpha }(\tau )\right) =\tau \end{aligned}$$

The above conditions should hold for any set of particular observations. Furthermore, if our theory shall not be considered refuted, we should have that

3 $$\begin{aligned} \Gamma _{\beta }\left( \phi (\varPi (\left\{ Obs\right\} _{\alpha })\right) \equiv \Gamma _{\beta }\circ \phi \circ \varPi \left( \left\{ Obs\right\} _{\alpha }\right) =\left\{ Obs\right\} _{\beta } \end{aligned}$$

i.e., that the theoretical conclusions elaborated by a set of observations with particularities $\alpha$ can be lifted to a corresponding set of observations $\left\{ Obs\right\} _{\beta }$ with particularity $\beta$. For the special case in which $\phi$ presents no elaboration (i.e., $\phi =Id$), Eq. (3) represents the standard requirement of reproducibility of experiments. The set of functions $\left\{ \phi \right\}$ are automorphisms of IW while the transformations $T_{\beta \alpha }$ among particular representations,

$$\begin{aligned} T_{\beta \alpha }=\Gamma _{\beta }\circ \varPi \left( \left\{ \cdot \right\} _{\alpha }\right) \end{aligned}$$

are automorphisms of SW.

It is important at this point to realise that:

- The triple $\left\{ \varPi ,\phi ,\Gamma \right\}$ can be designated with various (unfortunately ambiguous) names such as: schema and **theory**.
- The triple $\left\{ \varPi ,\phi ,\Gamma \right\}$ depends on the conjectures *h*, we are avoiding to overload the notation and omit the symbol. In no case we are going to mix symbols belonging to different theories (since every triple $\left\{ \varPi ,\phi ,\Gamma \right\} ^{h}$can be said to be the theory based upon the conjecture *h*).
- In the case of incorporating conjectures we will request for them to have a set of testable consequences larger than those that motivated their inclusion. If possible, they should be associated with a cognitive surpass (Piaget & García, 1989) [see also (Solari & Natiello, 2018)]. In terms of Kuipers (1999) the aims are: theory revision (aiming to empirical progress), truth approximation and generalisation/abstraction (the latter not in Kuipers). Notice that higher order theories are confronted with observations every time a particular lower order theory (associated to them in the dialectic universal-particular) is challenged. For example: we are testing logic almost all the time.
- It must also be kept in mind that $\left\{ \varPi ,\phi ,\Gamma \right\}$ depends not only on the observations but it depends as well on the question being asked. An example will help to grasp the idea. Assume we have harvested *m* Red Delicious apples, of large size but wormy, and *n* Granny Smith apples of small size and healthy. If we ask how many apples there are, we will project out the variety, size and sanitary status, keep only the quantity, use addition of integer numbers and write our answer as: there are $m+n$ apples, some Granny Smith, small and healthy and some Red delicious, large but wormy. However, not being experts in apples, we are inclined to believe that if the question is about the price of the lot we will have to keep all the attributes to make a prediction and we will probably fail anyway since we are not including information about the market. Moreover, to propose an expression to evaluate prices with the information at hand we will have to advance a formula, i.e., introduce a conjecture. In this example $\left\{ Obs\right\} _{\alpha }$ are the apples of the Sensible World, $\alpha$ denotes the characteristics of size, health and sort, which are projected out by $\varPi$, leaving us with an integer number representing the apples of the Ideal World of the theory. $\phi$ is integer addition and $\Gamma$ restores the characteristics projected out by $\varPi$.
- The elements of the triple are not independent and the theory is put into tension pulling from the ”extremes”. For a theory is constructed to answer some questions (the task of $\Gamma$) and data is procured to be plugged into a logical elaboration ($\phi$).^8^
- Probabilistic theories are not different in our respect from other theories. The idealisation of random events institutes that they have to be considered only collectively (as idealised by $\varPi$), individual events are instances of evaluation of random variables (in the $\Gamma$ version). We can only consider about them the set of allowed values and their probability distribution (in terms of frequencies). The logical and mathematical operations $\phi$ consent to elaborate answers from the premises in terms of probabilities. For example, quantum mechanics allows to predict the outcomes of experiments such as Stern-Gerlach’s that relates detector counts with different dispositions in the setup. Population dynamics represents the probable state of the population in terms of integer numbers and conciliates it with continuous time evolution resting on theorising about the evolution of probabilities of the population numbers.
- Peirce used abduction and retroduction indistinctly. Chiasson (2005) in his exegesis of Peirce argues that they should be distinguished as suggested by their etymological content, while “duction” means “lead” in latin “ab” stands for “away from” while “retro” means “going backward”. Thus, moving away from exegesis, the backward flow of error merits the name of retroduction. Some parts of it are abductive as they open up the reasoning for different possibilities like in the implication $\left\{ A,B\right\} \Rightarrow C$: when *C* evaluates to False, we have to admit that *A* or *B* are False, and we will have to confront at least three new hypothetical scenes. This means that the backward flow, the retroduction, calls for the formulation of new hypothesis as well as the collection of new kinds of data to discriminate among them, finally resulting in a more educated theory $\left\{ \varPi ,\phi ,\Gamma \right\} ^{*}$. This back and forth of construction, the iteration of successive reconstructions, has been presented in García (2011). The iteration stops when all predictions evaluate to True. We can then rest, believing in the theory. The iteration recommences when an anomaly is discovered and doubt urges us again. “Learning from errors” means overcoming failure. Hence, failure must be thanked for giving us an opportunity of learning. In contrast, success –stopping the iteration of doubts and beliefs– puts it on a pause, we stop learning when successful.
- The triple $\left\{ \varPi ,\phi ,\Gamma \right\}$ is a logical structure that allows to learn from mistakes. We discuss a historical example in Sect. 6
- Different theories may be compatible with the same observations. However, we are not comparing theories (or mixing them in any other form) but studying the process in which a given theory (under development) connects the Sensible World with the Ideal World back and forth. Statements (1) and (2) indicate the need to be consistent in terms of the production of ideas and their interpretation. Idealisation and interpretation are not independent of each other .

The structure arising from the triple $\left\{ \varPi ,\phi ,\Gamma \right\}$ corresponds to the idea of abduction-based reasoning, discussed in (Peirce, 1955, pp. 150–156). Let us consider it in more detail. The projection $\varPi$ suggests the nature of the relevant observations. It is supported by epistemological frames and other beliefs –and probably by some conjectures. It transforms observations into facts in the language of Piaget and García (1989). $\phi$ elaborates the ideation following the rules of mathematical logic and established theories and $\Gamma$ confronts the elaborated ideas against the sensible world. In this sense, $\Gamma$ can be called *interpretation*, connecting the ideas with the sensible world. If the confrontation is successful, we preliminarily accept the associated conjectures as satisfactory explanations. If, on the contrary, the theory is refuted, we abandon it and proceed to generate a new, improved, conjecture in the light of the refutation, which in turn means a new theory $\left\{ \varPi ,\phi ,\Gamma \right\} ^{*}$. This last process is illustrated by the backwards path in Fig. 1 indicating that disagreements between predictions and facts trigger improvements in $\varPi$. Also, if the theory does not contribute to organise and structure our views on the sensible world, it is rejected (it usually entails only to reject the conjectures). Conjectures that are impossible to test must be rejected as well (Peirce, 1955, pp. 150–151). Contemporary readers may recognise some of these ideas in Popper. Apart from precedence, Peirce goes further in his insight on $\varPi$.

Since there are different uses of the word “abduction” corresponding to different readings of Peirce, we will abound with another example. Consider an individual experiencing fever, headache and muscular ache. COVID-19 is the prevalent disease in his town these days. She consults a medical doctor who orders some tests: one that would detect signs of COVID-19, another detecting signs of flu and a third one detecting bacterial infections. Each of the hypotheses *h* proposes some projection $\varPi _{h}$, of the illness into the associated biochemistry. The tests are ordered because there is some logic and prior knowledge, $\phi$, that allows to elaborate each hypothesis into distinctive/discriminant biochemical outcomes for the test. The outcomes of the tests are considered confirmation (True) or rejection (False) of the various hypotheses. It is at this point, when the test is completed, that the process of abduction ends. It is not just $\varPi$. Nor it can be reduced to the guessing work performed (vulgar inference) even when the expectations on the hypothesis could be weighted by the current prevalence of illnesses. Probabilities might have biased our guessing work, but if we get a negative result for all the tests, we have learned something and our guessing work will be subsequently modified to produce new hypothesis compatible with the new information. In this respect, a proposition such as

Ignorance-preservation in the context of ignorance problems: Whereas deduction is truth-preserving and induction is probability-enhancing, abduction is ignorance-preserving (Gabbay & Woods, 2006)

indicates that either our approach of constructing scientific theories cannot be classified as an “ignorance problem” or that we associate a different meaning to “abduction”. It can easily be verified that at least the meaning of abduction is different. Let us emphasise the recursive, consistent and constant use of the method of analysis.

Considered as machines, humans are logic machines that control their own logic, which brings to mind the conclusion brought by Burks (1946) regarding Peirce’s thoughts on logical machines:

A computing machine starting from correct premises can arrive at correct conclusions. In what respects, then, does such a machine fail to infer? In the first place, the procedure of a machine does not have the element of conscious approval and control; it cannot certify the validity of its own inferences. Secondly, a computing machine lacks originality.

Neither $\varPi$ nor $\Gamma$ are deductive or inductive. Concerning $\Gamma$, it maps $IW\mapsto SW$, while induction goes in the same direction as $\varPi$ (namely $SW\mapsto IW$) and deduction connects $IW\mapsto IW$. Hence, $\Gamma$ is not induction since it has a different domain and it is not deduction since it has a different image. The only truly deductive part of the process lies in $\phi$. Science cannot be reduced to logic only. There are three types of reasoning (McAuliffe, 2015) in Peirce:

These three kinds of reasoning are Abduction, Induction, and Deduction. Deduction is the only necessary reasoning. It is the reasoning of mathematics. It starts from a hypothesis, the truth or falsity of which has nothing to do with the reasoning; and of course its conclusions are equally ideal ... Induction is the experimental testing of a theory. The justification of it is that, although the conclusion at any stage of the investigation may be more or less erroneous, yet the further application of the same method must correct the error. The only thing that induction accomplishes is to determine the value of a quantity. It sets out with a theory and it measures the degree of concordance of that theory with fact. It never can originate any idea whatever. No more can deduction. All the ideas of science come to it by the way of Abduction. Abduction consists in studying facts and devising a theory to explain them. Its only justification is that if we are ever to understand things at all, it must be in that way. (Peirce, 1994, CP 5.145)

The role of interpretation, as presented here, resembles the one attributed to “induction” in Peirce. The reason to change names is that most of us associate “induction” with a process that goes from observations to ideas, while interpretation goes in a somewhat opposite direction: From general ideas to particulars, as already explained.

**Afterthought on probabilities** While the present work is mostly concerned with the philosophical idea of science, this is, the science that belongs in IW, there are other forms of science pertaining to SW. The social use of science quite often rests on theories being currently developed, this is: in construction. In such cases, $\varPi$ and $\Gamma _{\alpha }$ are frequently based upon observations that do not fully explore the universe of particular situations. Thus, these theories have hidden variables outside the control of consciousness, or over-simplified relations between variables (laws). In such situations the predictions some times are correct and some times are incorrect. From the point of view of the construction of the theory the situation indicates incomplete understanding, and the theory is refuted; meaning that more research is needed. From a practical point of view, even an imperfect theory may have social value. In such cases, it will present its predictions in terms of probabilities.

**Afterthought on equivalence/inequivalence of theories** We say that two theories $\left\{ \varPi ,\phi ,\Gamma \right\}$ and $\left\{ \varPi ^{\prime },\phi ^{\prime },\Gamma ^{\prime }\right\}$ are equivalent if there exists *Q* invertible such that $Q\circ \varPi =\varPi ^{\prime }$ and $Q\circ \phi \circ \varPi =\phi ^{\prime }\circ \varPi ^{\prime }$, i.e., $\phi ^{\prime }\circ Q=Q\circ \phi$. This means that every idealised expression in one theory has a corresponding counterpart in the other theory. Further, we say that two theories explain all the observable (actually observed or potentially observable) if for every $\alpha$, $\Gamma _{\alpha }^{\prime }\circ \left( \phi ^{\prime }\circ \varPi ^{\prime }\right) =\Gamma _{\alpha }\circ \left( \phi \circ \varPi \right)$, i.e., all predictions in one theory correspond to a prediction in the other theory with the same outcome and tracing back to the same object in *SW*. Hence, it follows that

### Theorem 1

Two theories explaining all the observable (actually observed or potentially observable) are equivalent.

### Proof

Let $Q=\varPi ^{\prime }\circ \Gamma _{\alpha }$. By Eq. (1), $Q\circ \varPi =\left( \varPi ^{\prime }\circ \Gamma _{\alpha }\right) \circ \varPi =\varPi ^{\prime }$. Further, by Eq. (2) $Q\circ \phi \circ \varPi =\varPi ^{\prime }\circ \Gamma _{\alpha }\circ \left( \phi \circ \varPi \right) =\varPi ^{\prime }\circ \Gamma _{\alpha }^{\prime }\circ \left( \phi ^{\prime }\circ \varPi ^{\prime }\right) =\phi ^{\prime }\circ \varPi ^{\prime }$. That *Q* is invertible follows from noting that $Q^{-1}=\varPi \circ \Gamma _{\alpha }^{\prime }$. $\square$

### Corollary 1

Two theories are different (not equivalent) if there exists at least one experiment with different proposed/theorised outcome (be it that we expect different results or that the experiment can be explained in one theory but cannot be conceived in the other).

This result relates to the idea of “empirical equivalence” (Duhem, 1991) although here it is used in the opposite direction, namely illustrating that distinction among theories that exactly coincide in all observable tests is unnecessary and irrelevant, as far as appraisal of theories rests on the contrastation/refutation process. We advance here the result of the next subsection, namely that equivalent theories differ in arbitrary choices not relevant for the contrastation process (by Corollary 1) and that the *real *(what the rational support of the theory deals with) is what is left after eliminating arbitrariness.^9^

In the present context, as a consequence of Corollary 1, discriminating one equivalent theory from another on the basis of “Inference to the best [potential] explanation” (Boyd, 1983; Lipton, 2004) appears as subjective. Rather, preferring one equivalent theory to another reveals the kind of argumentation that scientists may consider more appealing. Apparently, this is what happened in the late 19th. century when physicists massively linked words such as “understanding” and “explaining” to one particular form of argumentation (see Sect. 3) rejecting other forms that were completely equivalent in terms of the experimental knowledge of their time.

### Subjective Formulations and the No Arbitrariness Principle

According to Peirce (1994, CP 6.522)

All our knowledge may be said to rest upon observed facts. It is true that there are psychological states which antecede our observing facts as such. Thus, it is a fact that I see an inkstand before me; but before I can say that I am obliged to have impressions of sense into which no idea of an inkstand, or of any separate object, or of an ” I,” or of seeing, enter at all; and it is true that my judging that I see an inkstand before me is the product of mental operations upon these impressions of sense. But it is only when the cognition has become worked up into a proposition, or judgment of a fact, that I can exercise any direct control over the process; and it is idle to discuss the ”legitimacy” of that which cannot be controlled. Observations of fact have, therefore, to be accepted as they occur.

Peirce found no reason to expect a relevant influence of such “psychological states” but such an idea must be reconsidered after Piaget (1999), because at least the notion of space, fundamental to physics, is a notion produced in the early development of the child. Then, the space, an a-priori of knowledge in Kant, needs to be accounted for. The map $\varPi$ represents the process of ideation. In the case of simple-ideation (Husserl) it is not consciously supervised, and then the resulting theories quite often carry some degree of arbitrariness. The *space* can be regarded as a convenient bookkeeping for the description of spatial relations; for each body it provides the spatial relation of the body with ego (the “observer”), thus it is a subjective view of spatial-relations immediately accessible to the intuition. Newton’s mechanics, for example, is of such quality. Such subjective formulation of theories are in the same relation with the real than the observable. Correspondingly, the schema of Fig. 1 applies as well.

It is at this point where the present discussion encounters Leibniz’ Principle of Sufficient Reason (Ballard, 1960). Weyl (2015) introduced an elaboration based upon it as the principle of symmetry in relation to the duality subjective-objective, yet he failed to identify properly the automorphism that had to be considered (Nozick, 1998; Catren, 2009; Fortin & Lombardi, 2015). The group of automorphisms that is relevant in this case is the *group of arbitrariness* with morphisms that map the expression of a natural law in terms of some arbitrary choices to the corresponding expression under a different arbitrariness. Such is the case of Cartesian space which is often prescribed by a reference point and orthonormal frame. The transformations between such reference frames correspond to the elements in the Euclidean group in three dimensions, $E^{3}$, consisting of the semi-direct product of translations and orthogonal transformations (reflections and rotations). Thus, for example, the spatial relations between *N* bodies (assumed point-like) can be written as set of 3*N* Cartesian coordinates, $R^{3N},$ yet what is real in it is the projection made by modding away the group of arbitrariness, $E^{3}$ (the Euclidean group), this is $R^{3N}/E^{3}$, Leibniz’ relational or *objective space*.

The requirement of objectivity (or intersubjectivity) imposes the composition of transformations to be associative (hence automorphisms), since e.g.,

4 $$\begin{aligned} T_{\beta \alpha }= & {} T_{\beta \delta }T_{\delta \alpha }=T_{\beta \delta }(T_{\delta \eta }T_{\eta \alpha }) =(T_{\beta \delta }T_{\delta \eta })T_{\eta \alpha } \end{aligned}$$

regardless of any particularity (“opinions” in the wording of Peirce) of $\delta ,\eta$. Hence,

#### Theorem 2

The transformations between arbitrary representations of SW form a group.

#### Proof

This group is called the *group of arbitrariness* of the theory. The existence of identity transformations follows from Eq. (1), that of inverse transformations from Eq. (2) and the equivalence in front of arbitrary particularities, while associativity follows from Eq. (4). $\square$

The No Arbitrariness Principle (NAP) reads (Solari & Natiello, 2018).

#### Principle 1

**[No Arbitrariness Principle (NAP)]** No knowledge of Nature depends on arbitrary decisions.

The standpoint we take is constructivist, NAP is a constructive principle for science, since we conceive science as an attempt to make a cosmos out of the chaos of the (sensory) world. This goal implies the higher conjecture that it is possible to achieve it. One of the main tools for moving ahead are cognitive surpasses which find unity (although at a more abstract level) of what appears as diverse and unrelated. Science then, can be viewed as a civilisational movement that continues the task in which each of us got involved as children (Piaget & García, 1989; Piaget, 1999).

Kuhn (1962) described normal science as somehow similar to “puzzle solving”. Notice that to solve a picture-puzzle it is required that all pieces fit together without conflict or violence, hence, in harmony. The collection of pieces put in harmony then revel a global picture that gives meaning to the individual pieces. However, this view somewhat contrasts with Kuhn (1962) when he states that “Normal science does not aim at novelties of fact or theory and, when successful, finds none.”

## The Concept of Science from Hertz to Einstein and Popper

Towards the second half of the 19th century, the force of the Enlightenment was declining in Europe while the second industrial revolution was developing. Important changes took place in philosophy, physics and mathematics.

The 19th century in philosophy experienced the entrance of materialism in the scene—with naturalism taking a dominant role after Darwin–, and its struggles with metaphysics. For the natural sciences began some sort of independisation from philosophy, a process that was intensified during the 20th century (Beiser, 2014). 

On the side of physics, the success of electromagnetic theory raised the issue of the nature of light and electromagnetic phenomena as opposed to mechanical phenomena. While the Newtonian grounds of mechanics seemed solid, instantaneous action at a distance could not be harmonised with the fact that electromagnetic phenomena appear to propagate with finite velocity. At the same time, the way of conceiving electrodynamic phenomena shifted from *hypotheses non fingo *(“I frame no hypotheses”)^10^ (Newton,^1687^, p. 506) to the *Bild* (Dieter, 1998; Heidelberger, 1998; Hoffmann, 1998; D’Agostino, 2004; Schiemann, 1998) concept (see below for a brief explanation).

In contrast with the motion of bodies addressed by mechanics, electromagnetic phenomena reach us mostly in a form not apt for being directly intuited. We do not see currents, for example, what we see is rather the deflection of a needle, while the current is inferred. Scientists as Lord Kelvin and Maxwell supported their thoughts with analogies. Lord Kelvin writes

I never satisfy myself until I can make a mechanical model of a thing. If I can make a mechanical model I can understand it. As long as I cannot make a mechanical model all the way through I cannot understand; and that is why I cannot get the electromagnetic theory. (Thompson, 2011, p.835).

while Maxwell says:

Now we are unable to conceive propagation in time, except either as the flight of a material substance through space, or as the propagation of a condition of motion or stress in a medium already existing in space.[...] If something is transmitted from one particle to another at a distance, what is its condition after it has left the one particle and before it has reached the other? ([866], Maxwell, 1873)

Indeed, light-travel is understood by analogies with bodies (we return to this idea in Sect. 5), still in our days, like a stone thrown by the source and captured by the detector. Also, electromagnetic waves were conceived mechanically and asked for a propagation medium that could sustain them after having abandoned the source and before reaching the detector.

A most decisive epistemological change was advanced by Hertz, a disciple of Helmholtz, who acted the idea that it is possible to separate the process of construction of a theory from the theory’s mathematical content. Regarding Maxwell’s electrodynamics, he states (Hertz, 1893, p. 21)

To the question: ‘What is Maxwell’s theory?’. I know of no shorter or more definite answer than the following:– Maxwell’s theory is Maxwell’s system of equations. Every theory which leads to the same system of equations, and therefore comprises the same possible phenomena, I would consider as being a form or special case of Maxwell’s theory;[...]

Hertz claimed that theories have to be provided with what he called *interpretation*, an element that runs alongside the mathematics and helps in constructing experiments related to theory (the action of $\Gamma$ in Sect. 2) while at the same time theory can be detached from its construction. Several inequivalent interpretations can, in this form, be attached to a theory. Hertz’ deep epistemological change has been highlighted by D’Agostino:

...by separating the mathematical structure of a theory from its modes of representation he [Hertz] has profoundly challenged the conception of a physical theory as an indivisible unity of the two – a conception accepted by Maxwell and other nineteenth century mathematical physicists.” D’Agostino (1968, 2004).

The Berlin school of Physics (see Footnote 4) made other profound contributions to epistemology. They introduced the *Bild* (Dieter, 1998; Heidelberger, 1998; Hoffmann, 1998; D’Agostino, 2004; Schiemann, 1998) approach, a form of understanding supported in images. As a brief explanation:

For Hertz, in contrast [with Helmholtz], representations of theories are signs of sensory impressions that are given to us. Only if we use theory to construct representations will it accomplish the most important task of natural knowledge, foresight of the future from experiences of the past. Heidelberger (1998)

Conceptually, since mental images are the outcome of sensorial perception, using images to organise understanding implies to habilitate sensorial based intuition to be used by analogy in other realms.

According to Poincaré, there was a crisis in mathematical physics by 1904 (Poincaré, 1913, original of September 1904). And indeed there was a deep crisis. The task of mathematicians at producing physical theories had been transferred to a new specialist: the theoretical physicist (Jungnickel & McCormmach, 2017) and furthermore, philosophers no longer exercised critical thinking in matters of science, at least in Germany. From Kant, through Reinhold, Fichte until Hegel, the prevalent movement considered that philosophy was “’the guardian of the sciences,’ their founder and systematizer” (Beiser, 2014, p. 15). But the movements that emerged after Hegel were mostly antihegelian. Peirce comments (from Lessons on the History of Science, ca. 1896):

German universities for a whole generation turned the cold shoulder to every man who did not extol their stale Hegelianism, until it became a stench in the nostrils of every man of common sense. Then the official fashion shifted, and a Hegelian is today treated in Germany with the same arrogant stupidity with which an anti-Hegelian formerly was. (Peirce,^1994^, CP 1.77)

For the new german philosophy the task of philosophy with respect to the sciences ranged from studying “the logic of the science” as acted by the scientists, to the extreme of the sciences declaring philosophy dead, as in “...neo-Kantian Jürgen Bona Meyer: ’The daughters now demand independence from their common mother, and they do not suffer it gladly when they are supervised or corrected; they would prefer that their old and morose mother lay herself to rest in her grave’” (quotations from (Beiser, 2014, pp. 17, 18).

Poincaré made an attempt to rescue some of the learnings of the old science (as he called it) in terms of principles that have been established by the old science and appeared to him as foundational. Among them the principle of relativity, the principle of minimal action, conservation of energy, Carnot’s 2nd law of thermodynamics and a few others. But he realised that these principles themselves were in crisis as well. It is interesting from the point of view of the present work to quote some of his words regarding the crisis of the principle of relativity (Poincaré, 1913, original in french of September 1904):

Let us pass to the principle of relativity: this not only is confirmed by daily experience, not only is it a necessary consequence of the hypothesis of central forces, but it is irresistibly imposed upon our good sense, and yet it also is assailed. Consider two electrified bodies; though they seem to us at rest, they are both carried along by the motion of the earth; an electric charge in motion, Rowland has taught us, is equivalent to a current; these two charged bodies are, therefore, equivalent to two parallel currents of the same sense and these two currents should attract each other. In measuring this attraction, we shall measure the velocity of the earth; not its velocity in relation to the sun or the fixed stars, but its absolute velocity.

I well know what will be said: It is not its absolute velocity that is measured, it is its velocity in relation to the ether. How unsatisfactory that is! Is it not evident that from the principle so understood we could no longer infer anything? It could no longer tell us anything just because it would no longer fear any contradiction. If we succeed in measuring anything, we shall always be free to say that this is not the absolute velocity, and if it is not the velocity in relation to the ether, it might always be the velocity in relation to some new unknown fluid with which we might fill space.

Here, Poincaré’s criticism is aligned with Peirce’s position, at least inasmuch he refuses to make non-refutable hypothesis.

The crisis identified by Poincaré proceeded with a new turn. By allowing free interpretations, the velocities involved in electromagnetism, that had been fully measurable relational velocities in Ampère, Faraday and Weber’s experiments as well as in Maxwell’s abduction, were reinterpreted in different ways. In their expressions of forces, and the derivation of them, Maxwell works with relative velocities between circuits (Maxwell, 1873). Lorentz (1892) reinterpreted relative velocities as absolute velocities referring to the ether in its expression of the electromagnetic force. Later Einstein (1905) kept Lorentz’ expression of the force while eradicating the ether, which implies another reinterpretation of the involved velocities as velocities with respect to an inertial frame, and proposing to use Lorentz’ transformations in place of Galilean boosts to restore the relativity principle. In so doing, he was loyal to Hertz epistemological point of view, changing the interpretation of the glyphs of Maxwell’s electrodynamics without changing the glyphs of the theory.

We have chosen Popper’s “Logic of Scientific Research” to represent the alternative to the epistemology schematically presented in Sect. (2) that evolved from the Berlin school. Popper’s view of the philosophy of sciences is well aligned with the post-Hegelians:

I suggest that it is the task of the logic of scientific discovery, or the logic of knowledge, to give a logical analysis of this procedure; that is, to analyse the method of the empirical sciences. (Popper, 1959, p. 4)

From pages 3 to 7 he address the problem of induction, concluding that induction is not the support of trust in science. He next writes against psychologism,

The initial stage, the act of conceiving or inventing a theory, seems to me neither to call for logical analysis nor to be susceptible of it. The question how it happens that a new idea occurs to a man— whether it is a musical theme, a dramatic conflict, or a scientific theory—may be of great interest to empirical psychology; but it is irrelevant to the logical analysis of scientific knowledge. (p. 7) [...]

returning recurrently to his main thesis:

According to the view that will be put forward here, the method of critically testing theories, and selecting them according to the results of tests, always proceeds on the following lines. From a new idea, put up tentatively, and **not yet justified in any way**—an anticipation, a hypothesis, a theoretical system, or **what you will**—conclusions are drawn by means of logical deduction. These conclusions are then compared with one another and with other relevant statements, so as to find what logical relations (such as equivalence, derivability, compatibility, or incompatibility) exist between them. (p. 9, emphasis added)

We have highlighted two expressions that give the clear impression that for Popper theories come from nowhere, leaving the process of production as not belonging to science. In short, for Popper the phenomenological moment appears as non-scientific.

By rejecting induction, branding other elements in the process of the construction of theories as psychologism and ignoring abduction in its original form he comes into terms with Einstein’s view

“Physics constitutes a logical system of thought which is in a state of evolution, and whose basis cannot be obtained through distillation by any inductive method from the experiences lived through, but which can only be attained by free invention. The justification (truth content) of the system rests in the proof of usefulness of the resulting theorems on the basis of sense experiences, where the relations of the latter to the former can only be comprehended intuitively. Evolution is going on in the direction of increasing simplicity of the logical basis”. Einstein (1940)

The Einstein-Popper view disregards $\varPi$, which is replaced by “free invention” and put outside science, this is, outside logical examination. As free invention, the replacement of a concept in a formulae by another one must be admitted, although its immediate consequence is that the new theory must be put to test from scratch [see (Popper, 1959, p. 63)]. Popper does not address how we go from glyphs into experiments, he apparently ignores $\Gamma$ as well. Einstein instead introduces “intuition” as part of the assessment of the “truth content”. This intuition shall not be confused with Husserl’s eidetic intuition that goes from the observed to the facts, for this one moves in the opposite direction, from theory/ideas into observations. In any case, to restore part of the coherence of the old science, such epistemology needs to be complemented with some principles such as the (intuited) Relativity principle and the requirement of reproducibility of experiments.

It must be noticed that the absence of logical conditions for the interpretation, as those that emerge from the abduction, makes free interpretation possible as well. The theories resulting from Popper’s epistemology ought to be considered less simple (in his terms) than those supported by the present (“pragmaticist”) approach since they can elude refutations by changing the interpretation, “saving” the core of mathematical relations.

## A “Pragmaticist” Criticism of Special Relativity

We want to illustrate in this section how the epistemological frame (Piaget & García, 1989)^11^ changes our appraisal of theories. We address the problem in practical terms considering Special Relativity, SR, one of the theories that prompted the need for a new epistemology as presented by Popper:

[Referring to other epistemological approaches] They will hardly be ready to grant this dignity to modern theoretical physics in which I and others see the most complete realization to date of what I call ‘empirical science’. [...] Thus I freely admit that in arriving at my proposals I have been guided, in the last analysis, by value judgments and predilections. (Popper, 1959, p. 15)

Poincaré (1905) introduced the Principle of relativity writing:

But it is not sufficient that the Euclidean (or non-Euclidean) geometry can ever be directly contradicted by experiment. Nor could it happen that it can only agree with experiment by a violation of the principle of sufficient reason, and of that of the relativity of space. Let me explain myself. Consider any material system whatever. We have to consider on the one hand the “state” of the various bodies of this system—for example, their temperature, their electric potential, etc.; and on the other hand their position in space. And among the data which enable us to define this position we distinguish the mutual distances of these bodies that define their relative positions, and the conditions which define the absolute position of the system and its absolute orientation in space. The law of the phenomena which will be produced in this system will depend on the state of these bodies, and on their mutual distances; but because of the relativity and the inertia of space, they will not depend on the absolute position and orientation of the system. In other words, *the state of the bodies and their mutual distances at any moment will solely depend on the state of the same bodies and on their mutual distances at the initial moment, but will in no way depend on the absolute initial position of the system and of its absolute initial orientation.* This is what we shall call, for the sake of abbreviation, the law of relativity. [Emphasis added]^12^

In Einstein (1905) the principle is presented as a conjecture, raised to the level of postulate:

The laws by which the states of physical systems undergo change are not affected, whether these changes of state be referred to the one or the other of two systems of co-ordinates in uniform translatory motion.

The relation between Poincaré version and Einstein’s can readily be seen from the quoted text. Einstein’s presentation of the law is operational. He dispenses of the intuitions and foundations presented by Poincaré. In particular, for Poincaré relative distances appear as real, a fundamental intuition, while in Einstein they will become apparent.

The present pragmaticist view indicates that the Principle of Relativity in classical mechanics is not a new or independent principle, but rather the consequence of requiring a rational foundation for our understanding and therefore eliminating arbitrariness. It is supported in the relational view of mechanics that goes back to Leibniz (Solari & Natiello, 2018) and we have called it the No Arbitrariness Principle (NAP). In some sense, it integrates Newton’s mechanics with Leibniz’ objections, thus extending beyond both. This form of surpass imposes that the mappings connecting presentations of dynamical processes under different arbitrary conditions constitute a *group* (a mathematical structure of associative binary operations having inverse and identity operation). In classical mechanics one of the groups relating arbitrary (subjective) choices is the group of Galilean coordinate transformations, eliminating the arbitrariness in the relative motion between reference systems. Relativity proposes to replace Galileo’s transformations by Lorentz transformations (LT). Under the pragmaticist schema it is then necessary that the LT’s constitute a group (which they don’t) and that they eliminate the corresponding arbitrariness in the relative motion between reference systems. Nothing is gained by enlarging these transformations with the full Poincaré-Lorentz group (PL), the arbitrariness cannot be fully eliminated.

The questions are: which is the residual arbitrariness that remains? How was it introduced in Special Relativity?

These questions examine the axioms and the inference (abduction) leading to the axioms, i.e., they concern the projection $\varPi$, before refutation or verification can enter the discussion. Therefore its criticism lies outside Popper’s epistemic approach and outside the “orthodox” approach (as described by Feigl) as well.

### On the Projection $\varPi$ Involved in Special Relativity

In the first paragraph of Einstein’s fundamental paper (Einstein, 1905) it is stated that physical phenomena depend on relative motion of the interacting parts, suggesting that relative velocity is a well-defined concept. This is a fundamental assumption of the theory that echoes the known properties of relative velocity in classical mechanics. This idea is completed in Part I, §3 stating that if a system *k* moves with velocity *v* with respect to a system *K*, and a system $K^{\prime }$ moves with velocity $-v$ with respect to *k*, then *K* and $K^{\prime }$ are at rest relative to each other. Finally, the change of coordinates between *k* and *K* is shown to be an invertible Lorentz Transformation (LT), let’s name it $L_{v}=L_{-v}^{-1}$.

In the terms of this work, relative velocity should be the outcome of $\varPi$ or a mathematical result constructed combining outcomes of $\varPi$, it should be in the image of the observable. Recall also that to be properly defined, relative velocity between e.g., two bodies *A* and *B* must be a quantity depending only on *A* and *B* without intervention of other observers, frames or references, a demand expressed by Eq. (4).

While in (Einstein, 1905) one of the systems is called “stationary” (meaning that *v* is the velocity of the “other” system as described by the stationary one), later Einstein (1907, p. 514) habilitates the LT’s as a change of (space-time) coordinates for systems in relative motion at constant velocity. Hence, according with the claim, for a reference frame $S_{0}$ plus a set of reference frames $S_{i},\ i\ge 1$ each one moving with velocity $u_{i}$ with respect to $S_{0}$ and all sharing the same origin of space-time coordinates, we have that $\left( x_{i},y_{i},z_{i},t_{i}\right) =L_{u_{i}}\left( x_{0},y_{0},z_{0},t_{0}\right)$. Since $L_{u_{i}}$is invertible, the transformation from reference frame $S_{i}$ to reference frame $S_{j}$ is $\left( x_{j},y_{j},z_{j},t_{j}\right) =\left( L_{u_{j}}\circ L_{-u_{i}}\right) \left( x_{i},y_{i},z_{i},t_{i}\right)$. According to the conjecture that the reference systems are of the same kind, there should be a relative velocity $u_{ji}$ so that its associated LT satisfies $\left( L_{u_{j}}\circ L_{-u_{i}}\right) =L_{u_{ji}}$. This is to say that the generalisation from Einstein (1905) to Einstein (1907) has the hidden hypothesis that the LT’s form a group, requiring that the successive applications of two such transformations is a LT as well. It is known that for any pair of velocities such that $u_{i}\times u_{j}\ne 0$ this is not the case, see for example (Silberstein, 1914; Gilmore, 1974). Hence, the hidden assumption must be rejected.

As a consequence, it is possible to put to experimental test Einstein’s 1905 theory in this regard, as for example is done in Doppler experiments (Dingle, 1960a; Kaivola et al., 1985; Mandelberg & Witten, 1962) with the theory surviving the test. In such case, the relative velocity must be computed using the standard methods of classical mechanics (since one of the systems is “stationary”).

On the other hand, to put the 1907 rewriting of the theory to this test it is required to show how the map $\varPi$ is used in order to compute the relative velocity between two bodies, say *A* and *B*, knowing their relative velocity as measured with respect to a third system *S*. Given the velocities $v_{AS}$ and $v_{BS}$ of *A* and *B* relative to *S* as initial data, there is no way to establish $v_{AB}$ by coordinate transformations as an outcome that does not involve *S*. The only candidate in the framework of Lorentz transformations (or even in the broader framework of the whole Poincaré-Lorentz group) would be $v_{AB}=v_{AS}\oplus \left( -v_{BS}\right)$ arising from the law of addition of velocities (Silberstein, 1914, p. 168), but (a) this quantity is intrinsically dependent on *S*, (b) it fails to satisfy the fundamental demand of reversibility, since $v_{AB}\ne -v_{BA}$ and (c) it is not unique. In short, there is no prescription on $\varPi$ that can relate the observable to the idealised under the indicated conjectures and the theory is not testable and should be rejected in terms of the epistemology proposed in this work.

## Maxwell and the Propagation of Light

Since the early times of optical science, the belief that light travels from source to detector at a finite velocity took form. The idea has its root in mechanical motion and suggested first the existence of a propagation medium and later of a “particle” named photon (sort of carrying the light to the target). This idea contrasts with e.g., Newtonian gravity, which is considered to plainly exist without further specification.

The body-like conception of light propagation is present in Maxwell, and lies as a support for the ether, that would be the medium through which light is travelling, in analogy with mechanical waves. While the quoted paragraph from Maxwell (see Sect. 3) supports the idea of the ether, Maxwell and also Faraday entertained doubts in this respect. The context of the quoted paragraph is to claim the right to investigate this physical hypothesis, strongly opposed by the Göttingen school that he much admired. In the same form, Maxwell wrote for and against the Göttingen approach. In what can be considered the most balanced expression, he writes

That theories apparently so fundamentally opposed should have so large a field of truth common to both is a fact the philosophical importance of which we cannot fully appreciate till we have reached a scientific altitude from which the true relation between hypotheses so different can be seen. (Maxwell, 2003, p. 228) [Address to the Mathematical and Physical Sections of the British Association. (Liverpool, September 15, 1870.)]

To his disappointment, the existence of the ether was refuted by experiments while the relational theories were abandoned by adherence to substantialism.

The body-like conception of light refers to an epistemological frame different and incompatible with the relational conception. A neat example of the relevance of the epistemological frame is Michelson-Morley’s experiment, that only makes sense if performed for electric disturbances travelling through the ether, opening for questions about velocities with respect to it. Within a relational view, this experiment makes little sense, since source and detector are at relative rest.

It is important to notice that Maxwell’s analogies using the ether produced some lasting contributions, like the “displacement current”. However, the displacement current can be produced without invoking the ether through the strategy of the Göttingen school: if there is evidence that electromagnetic disturbances propagate as waves, write it in formulae, i.e., build the corresponding phenomenological map. In contrast, Maxwell’s expression for the electromechanical force was rejected by experiments. The currently accepted electromagnetic force was produced by Lorentz arguing from the ether, but with a curiosity: the “virtual displacements” of the probe he used were at the same time displacements with respect to the ether and with respect to the remaining electromagnetic objects (Lorentz, 1892, §71). Hence, his argument holds true not because of the ether (call it free thinking) but because it once again agrees with the relational point of view. A recent discussion contains the details of the calculation.^13^

We defer to the Appendix further discussion of some of these points.

## On Abduction, Analogies and Electromagnetism

The history of electromagnetism is a very rich subject to learn about the epistemological changes in science at the end of the 19th century and the beginning of the 20th century. As electromagnetism, EM, posed higher difficulties than mechanics for understanding, scientists began to rest partially on analogies to produce conjectures to be tested by experiments first and later to promote the integration of EM laws. It is in this context that the idea of the ether irrupts and that finally the idea of fields was established. The process has been studied by Nersessian (1984). As we have explained and can be read in detail in Nersessian (1984) Faraday and Maxwell constantly indicated the advantages as well as the risks entailed in the use of analogies. They are good for suggesting conjectures, but the truth value of them must be established by other methods. Let us analyse from the present perspective Maxwell’s proposal.

To construct his set of equations Maxwell took into account experimental results, the principle of minimal action borrowed from mechanics and finally, the conjecture regarding the existence of the ether. Thus, the image of the observed by $\varPi$ is $\left\{ M,H\right\}$, where *M* represents the idealised experiments plus the mechanical principle, and *H* stands for hypothesis regarding the existence of the ether. The result of his mathematical elaboration, $\phi$, was a set of equations labelled from A to E (Maxwell 1873, [619]). The ether intervened by analogy in the production of the equation for the electromotive intensity (B), the mechanical force (C) and the equation for electric currents (E). We have then a set of intermediate conclusions given by $\left\{ A,\dots ,E\right\}$. Further elaboration from (B) and (E) (paragraph [783]) leads to the propagation of light as waves. Let *W* be the statement about the existence of waves and *F* the mechanical force. Then, we have the implications

$$\begin{aligned} \left\{ M,H\right\} \Rightarrow \left\{ A,\dots ,E\right\} \Rightarrow \left\{ W,F\right\} \end{aligned}$$

Contrastation with experimental results obtained interpreting the equations (application of $\Gamma$) supports the idea of *W* being true, but the evidence is opposed to *F*. Thus, *F* evaluates to False and the theory must be considered refuted: $\{W,F\}$ evaluates to False. Ignoring the intermediate steps, logic requires that we are in one of three possible cases: $\left\{ M,H\right\}$ evaluates to {False,True}, or {True, False} or {False, False}. Then, it is not possible to conclude that *M* is False.

Since later *H* was found to be False, consideration may move to $\{M,H^{*}\}$, using an alternative hypothesis $H^{*}$. Such an alternative (ether free) hypothesis had been advanced by Gauss (1870, bd.5 pp. 627–629,) in a letter to Weber of 1845, and was worked out by Lorenz (1867) to produce the same theory for EM waves as Maxwell’s. Later, Lorentz (1892) perfected Maxwell’s mechanical hypothesis, changing *M* by $M^{*}$ obtaining what is today known as the Lorentz’ force. Hence, there is a possible EM based upon $\left\{ M^{*},H^{*}\right\}$ which has not been explored.^14^ The historical course was to drop $\{M,H\}$ entirely and to select as starting points some, but not all, the equations in $\left\{ A,\dots ,E\right\}$, later adding the Lorentz force. In this form, the phenomenological work by Maxwell was left behind. Speaking of the field equations, Nersessian (2008, p. 20) says

...the field equations will not map back onto the domain from which the models through which Maxwell derived the equations were constructed.

On the other hand, for Nersessian (2008, p. 12)

Creative inference is often labeled “abduction”, but the nature of the inferential processes of abductive reasoning remains largely unspecified. Formulating an account of model-based reasoning provides a means of specifying the nature of the ampliative reasoning in abductive inference, for instance, analogy.

Notice that “Creative inference” is only a part of the abduction process as understood in the present work, but agrees with the use of “abduction” by other authors. If we have a problem *A* with properties $P_{A}$ and a second problem *B*, not clearly related, with properties $P_{B}\subset P_{A}$, then we have the right to guess that perhaps *B* has the properties in $P_{A}-P_{B}$ as well. After checking which $p\in (P_{A}-P_{B})$ are True, we can enlarge the set of properties of *B*, but unless every possible property that can be tested evaluates to True, the necessary conditions for equivalence of problem *B* with problem *A* are absent. Let $P_{B}^{*}$ be the augmented set of properties after the test. We might then decide that $P_{B}^{*}$ is a good set of axioms for our goals, perhaps with some additional assumptions/analogies that will be treated by iterating the procedure. The result is a patchwork that retains the truth content filtering out the expectations proved to be false. Such process goes against the unity of science and requires to leave the map $\varPi$ outside rational scrutiny. This approach is adopted by e.g., the Einstein-Popper epistemology (Sect. 3) and it is the result of the epistemological shift developed during the second half of the 19th century.

For a mind inclined to logic the sequence $A\Rightarrow B\Rightarrow \sim A$ simply speaks of inconsistency. Negating the expression we obtain $\sim A\Leftarrow (\sim B)\Leftarrow A$ thus both *A* and its consequences, *B* can be thought of as True and False at the same time. The set of premises *A* deserves to be called inconsistent. This is what happens with the derivation of electromagnetism using mechanical methods and the hypothesis of the ether: they cannot be made to stand together. We speculate that perhaps this problem lead Hertz to his bold remark opening the first of his two theoretical papers:

The system of ideas and formulae by which Maxwell represented electromagnetic phenomena is in its possible developments richer and more comprehensive than any other of the systems which have been devised for the same purpose. It is certainly desirable that a system which is so perfect, as far as its contents are concerned, should also be perfected as far as possible in regard to its form. The system should be so constructed as to allow its logical foundations to be easily recognised; all unessential ideas should be removed from it, and the relations of the essential ideas should be reduced to their simplest form. In this respect Maxwell’s own representation does not indicate the highest attainable goal; it frequently wavers between the conceptions which Maxwell found in existence, and those at which he arrived. (Hertz, 1893, p. 195)

Hertz decided then to sanitise the theory eliminating the potentials and, what is more important, modifying the equations to adapt them to the requisites of his understanding in terms of the *Bild* concept (see Sect. 3). The ether was a necessity of his form of understanding, the bodies have to drag the ether and body-like galilean invariance was required, and then imposed, modifying the equations.^15^

This is the form of the ether that, soon after, produced wrong insights and was proclaimed failed, we read:

That transparent bodies can move, without communicating their full velocity to the contained aether, was proven by Fizeau’s famous interference experiment with streaming water. (Lorentz, 1895, p. 1)

Are fields really in opposition with mechanics? For the Göttingen school and especially Ludwig Lorenz, the ether was never needed and fields were just a mathematical convenience (when appropriate). For them there is no space and therefore no need to discuss its properties. Only spatial relations are relevant.

For Hertz and many others that relied on analogies for understanding there was no other solution than reifying the space and dressing it with various fields. When Einstein announces in 1905 that the ether is not needed he is actually saying that the properties attributed to the ether can be directly attributed to the space.

In this sense, for Einstein the space is the ether:

When we speak here of aether, we are, of course, not referring to the corporeal aether of mechanical wave-theory that underlies Newtonian mechanics, whose individual points each have a velocity assigned to them. This theoretical construct has, in my opinion, been superseded by the special theory of relativity. Rather the discussion concerns, much more generally, those things thought of as physically real which, besides ponderable matter consisting of electrical elementary particles, play a role in the causal nexus of physics. Instead of ‘aether’, one could equally well speak of ‘the physical qualities of space’. [...]

It is usually believed that aether is foreign to Newtonian physics and that it was only the wave theory of light which introduced the notion of an omnipresent medium influencing, and affected by, physical phenomena. But this is not the case. Newtonian mechanics had its ‘aether’ in the sense indicated, albeit under the name ‘absolute space’.Einstein (1924)

It has been explained in several opportunities (Thomson, 1884; Lange, 1886; DiSalle, 1990, 2020; Solari & Natiello, 2021) that the idea of Newton’s mechanics resting on absolute space is alien to Newton. Mechanics rest upon “true motion” which is *not* motion in absolute space.

Synthesising our position: those artifacts needed to organise our thoughts belong with metaphysics. Analogies and imagination are great resources but need to be used with the caution advised by Faraday and Maxwell for otherwise they propitiate metaphysical artifacts.

## Concluding Remarks

We have discussed the logical requirements implied in the traditional conception of science and compared to new concepts socially dominant since the beginning of the 20th century. We have further shown than the traditional concept has a certain unifying power with respect to beliefs such as the requirement of reproducibility of experiments and symmetry requirements of scientific laws (in particular of physical laws). Additionally, the concept of science put forward is operative since it allows us to check the construction of theories [as it has been shown in Sect. (4)], implying as well that the sciences cannot be considered autonomous and much less independent of philosophy.

It is worth to be noticed that the schema of Fig. 1 operates not only at the level of completed theories but during the production of them as well. In our experience, the construction of theories implies the schema at every level of detail in what can be portrayed as a fractal structure. In the terms of Thagard (1978) the proposed schema is more *conciliar*, which is a decisive element in the evaluation of theories according to the programme of *Inference to the Best Explanation* (Lipton, 2004).

The replacement of the phenomenological map by free invention disproves any attempt of studying the fundamentals. For example, the relativity principle is accepted by habit or consensus without exerting any control on its appropriateness or on the conditions that it must satisfy. In such a form, the work of critical philosophy as a motion towards the fundamentals (as put by Hegel) is hindered, with the addition of gaining freedom for interpretations, thus making falsifiability harder to assess and helping to sustain dogmatic beliefs. Also, free invention made possible a continuous “progress” of science, without set backs, in as much as new interpretations and physical entities (if needed) are introduced, thus protecting the core beliefs.

The new form of science emerged in physics at the beginning of the 20th century and appears to evolve from a need for legitimating analogies. In this sense, anthropologist Sharon Traweek in her observation of the high-energy physicists community says:

Undergraduate physics students, to be successful, must display a high degree of intellectual skill, particularly in analogical thinking. The students learn from textbooks whose interpretation of physics is not to be challenged; in fact, it is not to be seen as interpretation. (Traweek, 1992, p. 74) [...] Teachers show students how to recognize that a new problem is like this or that familiar problem; in this introduction to the repertoire of soluble problems to be memorized, the student is taught not induction or deduction but analogic thinking. (p. 77)

The observation gives a fair idea on how this form of thinking is trained and selected. This shows how the epistemic frame is socially reproduced.

When abduction is conceived solely as the creative process of producing conjectures, analogy can be considered a form of abduction. However, when abduction is considered, as we do, the process of producing hypothesis, elaborating from them and confronting the results at empirical tests, concluding the abduction process by accepting unrefuted conjectures, analogies are only a possible mechanism in the proposing part of the process. However, analogies are associated more frequently to constructive processes within the Einstein-Popper epistemology.

The price to be paid when accepting analogies is high, as SR illustrates. We must leave behind universal time and relative positions accepting a new space-time. We have to accept a debilitated epistemology, hidden inconsistencies in the theory (the use of intuited velocities that cannot be measured) and we finally have to forbid natural questions concerning non-inertial frames. In contrast, if we accept that interactions are not bodies and hence, they do not need to have a place in space and consequently that analogies with bodies are of limited relevance, we not only maintain the unity of physics but strengthen it, showing that several seemingly independent principles all correspond to a conceptual unity [this is the property of being “conciliar” in Thagard (1978)]. The question is unavoidable: what did we obtain paying this price? We conjecture that the gains were social, but it deserves investigation.

With respect to Peirce’s complaint regarding that the word ”pragmatism” has been kidnapped, we observe that kidnapping words is a frequent practice. Currently most of those claiming to adhere to a relational view actually adhere to a formal procedure that has emptied the word of meaning, since instead of seeking for reality they make room for a world of incommensurate subjective opinions [see (Margenau & Mould, 1957) for a distinction between an “older meaning” and a “modern form” of relativity]. Even worse, the same can be said of ”critic” . How can science be critic when it does not allow to search for its fundamentals but considers only its consequences? In particular, the consistency of the triple $\left\{ \varPi ,\phi ,\Gamma \right\}$ is set aside, since both ends are debilitated or absent. Theories should be exposed to experimental analysis, but they may be rejected even earlier, if the phenomenological map is inadequate for the problem.

The phenomenological map connects and at the same time keeps as distinguished the world of ideas (IW) and the world of observations (SW). For critical philosophy, cognition requires both, as in Kant’s dictum:

Understanding cannot intuit, and the sensuous faculty cannot think. In no other way than from the united operation of both, can knowledge arise.

The phenomenological map that represents the dialogue between the sensuous faculty and our thoughts separates them and prevents hypostatisation, the reification of ideas. Not only Kant spoke against hypostatisation in his discussion of metaphysics. Faraday, a Natural Philosopher, wrote:

But it is always safe and philosophic to distinguish, as much as is in our power, fact from theory; the experience of past ages is sufficient to show us the wisdom of such a course; and considering the constant tendency of the mind to rest on an assumption, and, when it answers every present purpose, to forget that it is an assumption, we ought to remember that it, in such cases, becomes a prejudice, and inevitably interferes, more or less, with a clear-sighted judgment. (Faraday, 1844, p. 285)

It is not surprising then that simple considerations regarding the largely forgotten phenomenological map cast a distinctive light on our educated beliefs. Against the concept of science in Einstein and Popper (Sect. 3) we must raise Kant’s words (we invite the reader to substitute “pure reason” by “the sciences” in the next quotation)

Reason must be subject, in all its operations, to criticism, which must always be permitted to exercise its functions without restraint; otherwise its interests are imperilled and its influence obnoxious to suspicion. There is nothing, however useful, however sacred it may be, that can claim exemption from the searching examination of this supreme tribunal, which has no respect of persons. The very existence of reason depends upon this freedom; for the voice of reason is not that of a dictatorial and despotic power, it is rather like the vote of the citizens of a free state, every member of which must have the privilege of giving free expression to his doubts, and possess even the right of veto.

But while reason can never decline to submit itself to the tribunal of criticism, it has not always cause to dread the judgement of this court. Pure reason, however, when engaged in the sphere of dogmatism, is not so thoroughly conscious of a strict observance of its highest laws, as to appear before a higher judicial reason with perfect confidence. On the contrary, it must renounce its magnificent dogmatical pretensions in philosophy. (Kant, 1787, p. 475)

## Data Availability

Not applicable.

## A On Maxwell’s Argument Against the Göttingen Theory

In a communication to the Royal Society read June 18, 1868 (Maxwell, 2003, pp. 125–143), Maxwell comments and criticises the electromagnetic theories of Lorenz and Riemann. He would later in practice retract from this criticism in his treatise (Maxwell, 1873), admitting that Lorenz’ equations are equivalent to his system and, as we have seen in Sect. 5, also in a communication addressed to the Mathematical and Physical Sections of the British Association (Liverpool , September 15, 1870) (Maxwell, 2003, pp. 215–229). Nevertheless, it is interesting to use his argument to show how interpretation and analogy irrupts in scientific argumentation even in the words of a celebrated scientist. Maxwell writes (Maxwell, 2003, pp. 137–138): *For let two oppositely electrified bodies A and B travel along the line joining them with equal velocities in the direction AB, then if either the potential or the attraction of the bodies at a given time is that due to their position at some former time (as these authors suppose), B, the foremost body, will attract A forwards more than A attracts B backward.* Now let A and B be kept asunder by a rigid rod. The combined system, if set in motion in the direction AB, will pull in that direction with a force which may either continually augment the velocity, or may be used as an inexhaustible source of energy. I think that these remarkable deductions from the latest developments of Weber and Neumann’s theory can only be avoided by recognizing the action of a medium in electrical phenomena. (emphasis added) On a technical aspect, the “combined system” can be described easily with Lagrange’s formulation. It results in a Lagrangian independent of time (despite the retarded interaction, which is nullified by the rigid rod), meaning that Hamilton’s function is constant in time. With the introduction of generalised coordinates it does not require an explicit dependence on time. Hamilton’s function is the energy. The system does not create energy. Furthermore, since the interaction he considered was associated exclusively to Coulomb’s potential, the acceleration of the system is strictly zero and the potential is constant along the trajectory. The matter illustrates the dangers in “talking physics”.

The highlighted part deserves further analysis. Assume the described situation is possible without coercing it through a rod. The electrical action jumps to the space of the emitter and it delivers the action only when it is at a place in coincidence with the receiver (it sounds pretty much like a modern photon emitted from *A* towards *B*). Let $t_{B}$ be the time when the signal that left *A* is sensed by *B* and $t_{0}$ the time of emission. The distance travelled by this signal would be then $X_{B}(t_{B})-X_{A}(t_{0})$ [as measured in the frame of *A*, a subjective distance –see (Solari & Natiello, 2018)]. This coincides with the distance travelled by a body moving in a straight line from $X_{A}(t_{0})$ to $X_{B}(t_{B})$. In the same form, let $t_{A}$ be the time when the signal that left *B* at $t_{0}$ is sensed by *A*. The corresponding distance is then $X_{A}(t_{A})-X_{B}(t_{0})$ (in the frame of *B*). But since *B* is moving along the straight line *AB* while the signal is travelling, we have $|X_{B}(t_{B})-X_{A}(t_{0})|>|X_{A}(t_{A})-X_{B}(t_{0})|$. This relationship between positions, which is obvious for material bodies, is hence assumed to hold for electromagnetic interactions as well. We must admit that this form of thinking is odd: how do we know the signal jumps to the space of the receiver at the end of the process and not at the beginning? More relevant: this interpretation breaks the original objective conception of the Göttingen school in terms of relative positions and velocities. The relative distance between *A* and *B* is the same as that between *B* and *A*, except for orientation. We have broken the contract, and Eq. 1 is not satisfied, a tribute to the use of intuitions based upon images by analogy.

In any case, the observer that will measure the electromagnetic signal is associated to the detector and not to the emitter. Let us say that an array of detectors is placed in the frame of the receiver to track the electrical action. The receiver (observer) will register the distance between source and target at the time of emission. The action is tracked by the detectors of any observer since the moment of emission and on this basis we can compute the velocity of the signal. If $\tau$ is the time taken to reach the detector, we have $X_{B}(t_{0})-X_{A}(t_{0})$ which is an objective distance (equally registered from any reference system), as well as $\tau$, both invariant in classical physics. Then, $\frac{X_{B}(t_{0})-X_{A}(t_{0})}{\tau }$ is an invariant velocity and there is no problem involved. All observers determine the same value. The price paid is to leave analogies behind.

The “travelled distance” is a concept pertaining to absolute space. In contrast, we usually measure distances travelled by vehicles using an odograph. Place a car in a freight train and deliver it to some distant place. The odograph of the train will indicate a distance between train stops and the odograph of the car will indicate zero. We know both readings are correct, distances travelled are relative as well as velocities. To pretend that electromagnetic action has a speed solely determined by its identity is the same that asserting that light moves in absolute space

## Footnotes

1 See e.g., Standford Encyclopedia of Philosophy (Accessed 2020-04-23).

2 Perfection must be understood here in the sense of being faultless and capturing/creating the essence of the World. Ultimately, the two worlds can only be understood as one in front of the other, reciprocally defining themselves as in all the fundamental dialectic relations.

3 Feigl (1970). The ” orthodox” view of theories: remarks in defense as well as critique URL https://hdl.handle.net/11299/184651, retrieved from the University of Minnesota Digital Conservancy.

4 We leave to Sect. 3 a short explanation on how epistemology, particularly in physics, changed after the irruption in history of the Berlin school of physics with Helmholtz and Hertz among others. We designate as “traditional” the epistemological approach prevailing since Galileo until Maxwell (died 1879) and “new” the mature form that reached the evolution of the thoughts initiated at Berlin (to put a date, 1871 with Helmholtz becoming professor of physics). The transition time was then the epoch in which the belief in a material ether was prevalent.

5 We use particularities in the sense given by Peirce in the following expression: But observed facts relate exclusively to the particular circumstances that happened to exist when they were observed. They do not relate to any future occasions upon which we may be in doubt how we ought to act. They, therefore, do not, in themselves, contain any practical knowledge. (Peirce, 1994, CP 6.523)

6 The word “lift” takes in mathematics a related but different meaning in the context of fibre bundles (Rotman, 1988). Here $\varPi$ is a projection that produces an abstract idea from observations, an idea that fits them all, and can rightly be called “the essence” of the phenomena. Thus, in $\varPi :SW\mapsto IW$ we may regard *SW* as a sort of product space and the projected portion is the set of circumstances or arbitrariness, $\left\{ \alpha \right\}$. The image *IW* consists of the building blocks of the abduction process. Given the arbitrariness $\left\{ \alpha \right\}$ and *IW*, $\Gamma$ produces a reconstruction of *SW*. In mathematics, the lift would connect *SW* with $(IW,\left\{ \alpha \right\} )$. We use the word “lift” in a more colloquial form as the collection of inverses of $\varPi$ restricted to the observations with arbitrariness $\alpha$.

7 Hertz (Hertz (1893), p.20) makes this matter clear: Notwithstanding the greatest admiration for Maxwell’s mathematical conceptions, I have not always felt quite certain myself of having grasped the physical significance of his statements. Hence it was not possible for me to be guided in my experiments directly by Maxwell’s book. When testing theories, experimental scientists need $\Gamma$.

8 We often say ”data is born contaminated with theory”. Some things that are considered of no relevance are not measured (they are infinitely many, which is an excellent argument for not measuring them). At later times they may turn the experiment useless as their relevance becomes apparent.

9 We thank an anonymous reviewer for their insistence on giving consideration to alternative theories. The insight provided by the present approach was not consciously put in its construction. As such, it is equivalent to a prediction and shows, again, the conciliarity of our approach.

10 I frame no hypotheses; for whatever not deduced from the phenomena is to be called a hypothesis; and hypothesis, whether metaphysical or physical, whether of occult qualities or mechanical, have no place in experimental philosophy.

11 Generally speaking, an epistemological frame provides an a-priori form of organising concepts, formulating questions, producing and presenting answers. As such, it can discard questions as unsuitable or lacking interest and even produce “blindness” as in the example presented in García (1981). Moreover, the same question/answer might have completely different readings depending on the epistemological frame.

12 The paragraph illustrates as well the difficulties with the concept of “inertial systems”. Poincaré rests upon first conceiving absolute space and next suppressing its influence. Instead of saying that there are no such things as absolute position and absolute orientation, he writes “...because of the relativity and the inertia of space, they will not depend on the absolute position and orientation...”. Absolute space appears as an auxiliary metaphysical concept for thoughts, a first reference of space, that later will have to be suppressed. The discussion of the concept of inertial systems is part of the transition from classical to current physics (Solari & Natiello, 2021).

13 M. A. Natiello and H.G. Solari, Relational electromagnetism and the electromagnetic force https://arxiv.org/abs/2102.13108.

14 We claim that the resulting theory differs only in some interpretation from the currently accepted theory but it is more conciliar. Solari and Natiello (2022).

15 Notice that since we are speaking of actions and not bodies, we have to ask first for the form in which the subjective view of the space must be removed in this case rather than acting by analogy. This is, Hertz motivation is correct but the resolution requires a cognitive surpass not analogy.
